# Smoking Aggravates Inflammation, Fibrogenesis, Angiogenesis and Cancer Risk in Patients With Cirrhosis

**DOI:** 10.1111/liv.70314

**Published:** 2025-09-03

**Authors:** Nina Dominik, Benedikt Simbrunner, Bernhard Scheiner, Michael Schwarz, Lukas Hartl, Mathias Jachs, Lorenz Balcar, Georg Semmler, Georg Kramer, Christian Sebesta, Michael Trauner, Matthias Pinter, Mattias Mandorfer, Thomas Reiberger, Benedikt Silvester Hofer

**Affiliations:** ^1^ Division of Gastroenterology and Hepatology, Department of Medicine III Medical University of Vienna Vienna Austria; ^2^ Vienna Hepatic Hemodynamic Lab, Division of Gastroenterology and Hepatology, Department of Medicine III Medical University of Vienna Vienna Austria; ^3^ Christian Doppler Lab for Portal Hypertension and Liver Fibrosis Medical University of Vienna Vienna Austria

**Keywords:** angiogenesis, cirrhosis, fibrogenesis, HCC, inflammation, smoking

## Abstract

**Background and Aims:**

Smoking induces a proinflammatory state, yet its role in advanced chronic liver disease (ACLD) remains understudied. This study evaluated its impact on disease‐driving mechanisms and clinical outcomes in ACLD patients.

**Methods:**

ACLD patients undergoing hepatic venous pressure gradient measurements from 2017 to 2021 were included. The association of smoking with biomarkers of inflammation, fibrogenesis, angiogenesis and the incidence of hepatocellular carcinoma (HCC), extrahepatic malignancies and mortality was examined.

**Results:**

Among 339 ACLD patients (66.1% men, median age: 56.8 years, MELD: 11, HVPG: 17 mmHg), 62% (*n* = 210) were ever‐smokers (*n* = 78 former, *n* = 132 active). Compared to never/former smokers, active smokers exhibited significantly higher white blood cell counts (5.49 vs. former: 4.74 vs. never: 4.25 G/L; *p* < 0.001) and C‐reactive protein levels (CRP: 0.36 vs. former: 0.29 vs. 0.21 mg/dL; *p* = 0.035). Active smokers showed upregulated fibrogenic (TIMP‐1: 364 vs. former: 324 vs. never: 278 ng/mL, *p* < 0.001; P3NP: 20.9 vs. former: 15.6 vs. never: 17.2 μg/L, *p* = 0.008) and angiogenic (PLGF: 21.7 vs. former: 18.2 vs. never: 19.0 pg/mL, *p* = 0.004) activity markers. Over a median follow‐up of 30.4 months, ever‐smokers exhibited a higher incidence of extrahepatic malignancies (SHR: 11.30; *p* = 0.019) and numerically higher HCC incidence (SHR: 2.27 *p* = 0.150; mostly evident in Child‐Pugh A patients: SHR: 7.44, *p* = 0.053). All‐cause or liver‐related mortality risk did not differ significantly between ever‐smokers and never‐smokers.

**Conclusion:**

Smoking is linked to an upregulation of inflammatory, fibrogenic and angiogenic processes in ACLD and increases the risk of extrahepatic malignancies. These findings underscore the importance of strategies supporting smoking cessation in ACLD patients.

**Trial Registration:**

NCT03267615

**Summary**

- Our research highlights the detrimental impact of smoking on patients with advanced chronic liver disease (ACLD).
- We demonstrate that smoking is linked to an upregulation of inflammatory, fibrogenic and angiogenic processes in ACLD and increases the risk of extrahepatic malignancy.
- These findings underscore the critical imperative for smoking cessation as a cornerstone in managing ACLD progression and improving patient outcomes.

**Abbreviations**
ACLD: Advanced Chronic Liver Disease
ACLF: Acute‐on‐Chronic Liver Failure
ALD: Alcohol‐Related Liver Disease
CRP: C‐Reactive Protein
ELF: Enhanced Liver Fibrosis
HCC: Hepatocellular Carcinoma
HVPG: Hepatic Venous Pressure Gradient
INR: International Normalised Ratio
LBP: Lipopolysaccharide Binding Protein
sVEGFR1: Soluble vascular endothelial growth factor‐receptor 1
MACE: Major Adverse Cardiovascular Events
MASLD: Metabolic Dysfunction‐Associated Steatotic Liver Disease
MELD: Model for End‐Stage Liver Disease
NSBB: Nonselective Beta Blocker
PLGF: Placental Growth Factor
P3NP: Procollagen Type III N‐Terminal Propeptide
PY: Pack Years
TIMP‐1: Tissue Inhibitor of Metalloproteinases 1
WBC: White Blood Cell Count

## 1 Introduction

In 2015, tobacco smoking was the second leading risk factor for premature death and disability worldwide and has claimed over 5 million lives since 1990 [1]. Simultaneously, the burden of chronic liver disease, driven by factors including alcohol consumption, obesity and viral hepatitis, is increasing worldwide [2].

Importantly, smoking and chronic liver disease often coexist, as close to 40% of patients with advanced chronic liver disease (ACLD) are active or former smokers [3, 4, 5]. The prevalence is particularly high in patients with alcohol‐related liver disease (ALD) with smoking rates being three times higher than in the general population [6].

Currently available clinical evidence suggests that smoking negatively affects chronic liver disease by promoting hepatocarcinogenesis [7, 8, 9] and stimulating hepatic fibrogenesis [10, 11]. However, the impact of smoking on liver‐related outcomes is multifaceted. In a systematic review of mortality predictors in ACLD patients, smoking was identified as an independent predictor of survival [12]. Consistent with this, smoking has also been shown to increase mortality in patients with metabolic dysfunction‐associated steatotic liver disease (MASLD) in prospective population‐based studies [13, 14]. However, some studies have found that smoking is only a minor risk factor for first hepatic decompensation and a weak predictor of 6‐month mortality after hospital discharge in decompensated patients [15, 16], highlighting the need to assess the impact of smoking in both compensated ACLD, where liver function is still preserved, and decompensated ACLD separately.

Smoking has been identified as a key risk factor for hepatocellular carcinoma (HCC) in numerous studies and meta‐analyses [7, 8, 9, 17], yet the underlying pathomechanisms remain ill‐defined. Chemicals found in tobacco are metabolised into carcinogenic molecules in the liver [18] and smoking is associated with immunosuppression and telomere dysfunction, two early mechanisms in cancer development [19, 20]. Additionally, experimental evidence shows that inflammation plays a crucial role in fostering a tumour‐promoting microenvironment [21]. The presence of benzene—a human carcinogen that is metabolised in the liver to toxic phenols and hydroquinone—in cigarette smoke has been shown to induce oxidative stress and DNA damage in the liver [22, 23]. Since benzene exposure is widely recognised as a cause of extrahepatic malignancies, such as leukaemia [24], it likely contributes to smoking‐induced liver injury and hepatocarcinogenesis, thereby increasing the risks of fibrosis and hepatocellular carcinoma in ACLD patients who smoke.

Profibrogenic effects of smoking in the liver have been suggested by previous studies showing that nicotine exposure can induce the activation of hepatic fibroblasts and profibrogenic pathways [10, 11], as also reported for other organs [11]. Other potential mechanisms involve the induction of proinflammatory cytokine secretion and inflammatory cell recruitment [25] as well as reactive oxygen species production, that is, oxidative stress, leading to hepatocellular damage [26].

Despite the known risks of smoking, its role in ACLD remains underexplored, likely overshadowed by the perceived ‘dominant’ risk associated with advanced chronic liver disease [2]. This study aims to provide a clearer understanding of the influence of smoking on key liver disease and cancer driving mechanisms as well as on hepatic and extrahepatic cancer rates and mortality in well‐characterised ACLD patients.

## 2 Methods

### 2.1 Study Population and Study Design

In this single‐centre study, patients with ACLD undergoing a measurement of the hepatic venous pressure gradient (HVPG) in accordance with a standardised protocol [27] at the Hepatic Hemodynamic Lab at the Vienna General Hospital between January 2017 and December 2021 were included.

Inclusion criteria were: (i) age ≥ 18 years, (ii) a verified diagnosis of ACLD, (iii) signed written informed consent and (iv) sufficient information regarding smoking status and packyears (PY). In accordance with national [28] and international [29] guidelines, the diagnosis of ACLD was either based on liver biopsy confirming advanced fibrosis/cirrhosis, a liver stiffness measurement ≥ 10 kPa or an HVPG > 5 mmHg. Exclusion criteria were: (i) prior liver transplantation or transjugular intrahepatic shunt insertion, (ii) active or prior hepatocellular carcinoma (HCC), (iii) active extrahepatic malignancy, (iv) portal vein thrombosis, (v) acute decompensation, active infection or acute‐on‐chronic liver failure (ACLF), (vii) nonselective beta blocker (NSBB) therapy at the time of measurement or (viii) insufficient data quality regarding baseline or follow‐up.

This study follows a hybrid design, with a retrospective assessment of smoking history at baseline and a prospective evaluation of clinical outcomes.

Smoking status and PY were determined from electronic health records based on at least two independent reports to optimise accuracy and minimise recall bias. Smoking status was determined at baseline, and cessation dates documented in our records pertained to cessation prior to study inclusion. Any changes in smoking behaviour during follow‐up were not assessed. To assess the potential impact of prior smoking habit modification, we conducted analyses evaluating the duration of smoking cessation in former smokers and its association with biomarkers and clinical outcomes.

All patients were systematically followed from the time of HVPG measurement onward, ensuring a prospective evaluation of primary outcomes, including MACE, HCC, extrahepatic malignancies and mortality. Routine blood samples, including those for biomarker analysis, were collected at baseline and analysed at the ISO‐certified Department of Laboratory Medicine of the Vienna General Hospital, following standardised procedures.

### 2.2 Clinical Follow‐Up and Events of Interest

Patients were followed up prospectively at the liver cirrhosis outpatient clinic of the Vienna General Hospital. Furthermore, all inpatient stays during the follow‐up period were documented and analysed. The primary outcome parameters of interest were (i) hepatic and extrahepatic malignancies, (ii) all‐cause and liver‐related mortality and (iii) major cardiovascular events (MACE) including myocardial infarction, stroke and cardiovascular death. Death was considered liver‐related when occurring as a direct consequence of the underlying liver disease, including acute‐on‐chronic liver failure (ACLF), HCC‐related mortality and major complications of portal hypertension such as variceal bleeding, spontaneous bacterial peritonitis (SBP) or hepatorenal syndrome [29]. Data regarding the cause and date of death were acquired from the patient's electronic health records and from a query to the national death registry (Statistik Austria).

### 2.3 Statistical Analysis

Statistical analyses were conducted using R 4.3.1 (R Core Team, R Foundation for Statistical Computing, Vienna, Austria). Continuous variables are reported as median and interquartile range. Two group comparisons were conducted using an independent samples *t*‐test or a Mann–Whitney U test, as applicable. For multiple groups, comparisons of continuous variables were performed using an analysis of variance with Tukey's multiple comparisons correction or a Kruskal–Wallis test with Bonferroni correction, as applicable. The presence of a normal distribution was assessed by visual inspection of density plots and the Shapiro–Wilk test. The correlation of two continuous variables was analysed based on the Spearman's rank correlation coefficient. Multiple linear regression models, including MELD and HVPG, modelled using restricted cubic splines with three degrees of freedom, were employed to account for the severity of liver dysfunction and portal hypertension. Categorical variables were reported as number (percentage) and compared by Pearson's Chi‐squared test or Fisher's exact test.

The prognostic impact of smoking on MACE, HCC, extrahepatic malignancies, all‐cause mortality and liver‐related death was analysed using uni‐ and multivariable cause‐specific Fine and Gray competing risk regression models. Death and liver transplantation were treated as competing events within the analysis of MACE, HCC and extrahepatic malignancies. Non‐liver–related death and liver transplantation were considered as competing events within the analysis of liver‐related mortality. For all‐cause mortality, liver transplantation was treated as a competing event. Within outcome models, patients were followed from the time of HVPG measurement until the earliest occurrence of the event of interest, a competing event, or the date of last follow‐up. Multivariable models were adjusted for relevant cofactors in three separate models: (i) Model 1 included MELD (Model for End‐Stage Liver Disease; United Network for Organ Sharing MELD score [2016]) and HVPG; (ii) Model 2 included age and sex; (iii) Model 3 included diabetes and viral aetiology of liver disease. The presence of multicollinearity was evaluated by variance inflation factor analysis. Outcome analyses were depicted using cumulative incidence plots. The median follow‐up time of the study was calculated using the reverse Kaplan–Meier method. Two‐sided *p* values < 0.05 were considered statistically significant.

### 2.4 Ethical Considerations

All analyses were conducted in accordance with the 1964 Declaration of Helsinki and its later amendments, the Declaration of Istanbul and approved by the local ethics committee of the Medical University of Vienna (1262/2017). All patients signed an informed consent form prior to study inclusion.

## 3 Results

### 3.1 Patient Cohort

During the study period, 625 ACLD patients with known smoking status underwent a reliable HVPG measurement, with 286 patients presenting at least one exclusion criterion. Thus, 339 patients were included in the final study cohort. The detailed patient selection process is shown in Figure S1.

Of all included patients, 66.1% were male and the median age was 56.8 (49.1–64.7) years. The median HVPG was 17 (12–20) mmHg, the median MELD was 11 (9–15) and 52.5%, 36.0% and 11.5% of patients were classified as Child‐Pugh stage A, B and C at baseline respectively. At baseline, 38.9% presented without prior hepatic decompensation. With regard to smoking status, 129 patients (38.1%) were classified as never‐smokers and 210 (61.9%) as ever‐smokers, with 78 (23.0%) former smokers and 132 (38.9%) active smokers. With a median age of 62.4 years at study inclusion, former smokers were significantly older than never‐smokers (54.0 years) and active smokers (54.4 years; *p* < 0.001). The proportion of male patients was significantly higher in former (75.6%) and active (72.0%) smokers than never‐smokers (54.3%; *p* = 0.001). A detailed overview of all baseline characteristics is shown in Table 1.

**TABLE 1:** Patient characteristics stratified by smoking status.

	Full cohort *n* = 339	Never smokers *n* = 129	Former smokers *n* = 78	Active smokers *n* = 132	*p*
Age, years	56.8 (49.1–64.7)	54.0 (46.5–65.2)^a^	62.4 (56.4–68.6)*, ^b^	54.4 (45.6–61.0)^a^	**< 0.001**
Sex, male	224 (66.1%)	70 (54.3%)	59 (75.6%)	95 (72.0%)	**0.001**
Aetiology					**< 0.001**
ALD	158 (46.6%)	50 (38.8%)	33 (42.3%)	75 (56.8%)	
Viral	56 (16.5%)	23 (17.8%)	9 (11.5%)	24 (18.2%)	
ALD/Viral	21 (6.2%)	0 (0%)	5 (6.4%)	16 (12.1%)	
MASLD	30 (8.8%)	14 (10.9%)	14 (17.9%)	2 (1.5%)	
Cholestatic	20 (5.9%)	13 (10.1%)	4 (5.1%)	3 (2.3%)	
Other	54 (15.9%)	29 (22.5%)	13 (16.7%)	12 (9.1%)	
HVPG, mmHg	17 (12–20)	16 (11–20)	17 (10–21)	17 (13–21)	0.450
Compensated	132 (38.9%)	61 (47.3%)	31 (39.7%)	40 (30.3%)	**0.019**
MELD	11 (9–15)	11 (8–15)	12 (9–16)	10 (9–15)	0.360
Child‐Pugh stage					0.861
A	178 (52.5%)	69 (53.5%)	41 (52.6%)	68 (51.5%)	
B	122 (36.0%)	48 (37.2%)	26 (33.3%)	48 (36.4%)	
C	39 (11.5%)	12 (9.3%)	11 (14.1%)	16 (12.1%)	
Diabetes	87 (25.7%)	35 (27.1%)	30 (38.5%)	22 (16.7%)	**0.002**
Art. hypertension	109 (32.2%)	45 (34.9%)	38 (48.7%)	26 (19.7%)	**< 0.001**
Albumin, g/L	36.7 (32.6–40.2)	36.8 (32.8–40.2)	36.5 (32.5–40.2)	36.7 (32.8–40.2)	0.873
Bilirubin, mg/dL	1.06 (0.72–1.96)	1.12 (0.75–1.95)	1.14 (0.75–1.96)	0.97 (0.67–1.94)	0.242
INR	1.3 (1.2–1.5)	1.3 (1.2–1.6)	1.3 (1.2–1.5)	1.3 (1.2–1.5)	0.920
Creatinine, mg/dL	0.75 (0.61–0.95)	0.73 (0.59–0.90)^a^	0.82 (0.66–1.07)*, ^b^	0.72 (0.60–0.88)^a^	**0.009**
Sodium, mmol/L	138 (136–141)	139 (137–141)	138 (135–140)	139 (136–140)	0.077
Platelets, G/L	102 (75–142)	100 (76–132)	99 (78–138)	106 (70–154)	0.649
WBC, G/L	4.78 (3.30–6.35)	4.25 (3.03–5.52)^b^	4.74 (3.20–5.93)^b^	5.49 (3.90–7.30)*, ^a^	**< 0.001**
CRP, mg/dL	0.29 (0.12–0.68)	0.21 (0.11–0.59)	0.29 (0.13–0.67)	0.36 (0.17–0.75)	**0.035**
IL‐6, mg/dL	9.36 (5.27–17.7)	9.61 (4.73–17.2)	9.80 (5.47–18.8)	8.67 (5.66–17.0)	0.519
LBP, μg/mL	6.89 (5.20–8.89)	6.20 (4.92–8.49)	7.32 (5.33–8.95)	7.12 (5.45–9.40)	0.080
vWF Antigen, %	273 (210–354)	264 (205–333)	274 (208–349)	287 (216–365)	0.420
AST, U/L	42 (30–58)	43 (32–59)	41 (29–60)	42 (29–56)	0.889
ALT, U/L	30 (21–43)	32 (23–46)	31 (21–43)	27 (19–40)	0.073
ELF	11.4 (10.4–12.4)	11.1 (10.3–12.2)	11.5 (10.6–12.3)	11.6 (10.8–12.8)	0.090
TIMP‐1, ng/mL	325 (245–467)	278 (211–394)^b^	324 (252–434)	364 (275–501)*	**< 0.001**
P3NP, μg/L	18.0 (11.4–30.5)	17.2 (10.6–28.7)^b^	15.6 (10.9–24.9)^b^	20.9 (13.6–35.0)*, ^a^	**0.008**
HA, ng/mL	223 (108–468)	179 (96–365)	249 (115–414)	234 (115–540)	0.266
sVEGFR1, pg/mL	106 (91–127)	106 (93–121)	103 (91–125)	107 (89–135)	0.847
PLGF, pg/mL	19.8 (16.3–26.0)	19.0 (14.8–22.5)^b^	18.2 (15.4–25.3)^b^	21.7 (17.9–29.1)*, ^a^	**0.004**

*Note:* Data presented as number *n* (%) or median (IQR). Data on TIMP‐1, P3NP and HA as components of the ELF test are missing in *n* = 20 patients. Data on sFLT1 (sVEGFR1) and PLGF are missing in *n* = 153 patients. *p* values in bold indicate statistical significance.

Abbreviations: ALD, alcohol‐related liver disease; ALT, alanine aminotransferase; AST, aspartate aminotransferase; CRP, C‐reactive protein; ELF enhanced liver fibrosis test; HA, hyaluronic acid; HVPG, hepatic venous pressure gradient; IL‐6, interleukin 6; INR, international normalised ratio; LBP, lipopolysaccharide binding protein; MASLD, metabolic‐dysfunction associated steatotic liver disease; MELD, model for end‐stage liver disease; P3NP, procollagen type III N‐terminal propeptide; PLGF, placental growth factor; sVEGFR1, soluble vascular endothelial growth factor receptor 1; TIMP‐1, tissue inhibitor of metalloproteinases 1; vWF, von Willebrand factor; WBC, white blood cell count.

* *p* < 0.05 when compared to never smokers.

^a^ Versus former smokers.

^b^ Versus smokers.

The median number of PY in ever‐smokers was 30 (19–40), with 30 (15–45) in former smokers and 30 (20–40) in active smokers. 53.8% of ever‐smokers had a history of at least 30 PY, while only 8.6% reported less than 10 PY. In 78 former smokers, the median duration of smoking cessation prior to study inclusion was 12.7 years (IQR: 5.4–19.5 years). At baseline, 21% of former smokers (*n* = 16) had quit within the last 3 years, 23% (*n* = 18) had quit between 3 and 10 years prior and 56% (*n* = 44) had been smoke‐free for more than 10 years. During the follow‐up period, no active smokers at baseline reported smoking cessation for at least 6 months.

The most prevalent underlying aetiologies were ALD in 46.6% and viral liver disease in 16.5%, while 6.2% had combined alcohol‐related and viral liver disease. Among patients with viral (co‐)aetiology, *n* = 19 (33.9%) were viraemic at enrolment, while *n* = 39 (69.6%) had achieved sustained virologic response (SVR) through antiviral therapy (for a median of 2.4 years) prior to enrolment. Across different aetiologies, ALD patients showed the highest prevalence of former/active smokers with 68.4%, compared to 58.9% in viral liver disease, 53.3% in MASLD and 35.0% in cholestatic liver diseases. Among the 158 patients with ALD aetiology, 101 (63.9%) reported abstinence for at least 6 months at baseline, while 57 (36.1%) reported occasional or ongoing alcohol consumption. When stratified by baseline smoking status within the ALD cohort, ongoing alcohol consumption at baseline was reported by a significantly higher proportion of active smokers (45.3%, *n* = 34/75) compared to former smokers (33.3%, *n* = 11/33) and never‐smokers (24.0%, *n* = 12/50) (*p* = 0.043). During follow‐up, relapse or continued alcohol consumption was documented in 52.0% (*n* = 39/75) of active smokers with ALD, which was a significantly higher rate than observed in former smokers (36.4%, *n* = 12/33) and never‐smokers (30.0%, *n* = 15/50) within the ALD cohort (*p* = 0.033).

### 3.2 Impact of Smoking on Disease‐Driving Mechanisms in ACLD

While the number of patients without prior hepatic decompensation decreased gradually from 47.3% in never‐smokers to 39.7% in former smokers and 30.3% in active smokers, there were no significant differences in MELD, Child‐Pugh stage, or HVPG at baseline (Table 1).

Regarding systemic inflammation, active smokers showed a significantly higher white blood cell count (WBC) (5.49 G/L) compared to former smokers (4.74 G/L) or never‐smokers (4.25 G/L; *p* < 0.001). Similarly, C‐reactive protein (CRP) was highest in active smokers (0.36 mg/dL vs. former: 0.29 mg/dL vs. never: 0.21 mg/dL; *p* = 0.035). For lipopolysaccharide binding protein (LBP), median levels were markedly higher in active (7.12 μg/mL) and former (7.32 μg/mL) smokers as compared to never‐smokers (6.20 μg/mL; *p* = 0.080) (Table 1, Figure S2). After including MELD and HVPG in multiple linear regression models, active smokers showed significantly higher WBC (*p* < 0.001), CRP (*p* = 0.015) and LBP (*p* = 0.029) levels compared to never‐smokers, while no significant differences were found between former and never‐smokers. When analysing differences according to PY in active and former smokers, no significant differences in WBC, CRP or LBP were observed between < 30 and ≥ 30 PY (Table S1).

With regard to fibrogenesis, there was a stepwise increase in the median ELF test levels from 11.1 in never‐smokers to 11.5 in former smokers and 11.6 in active smokers (*p* = 0.090). For distinct components of the ELF test, active smokers showed significantly higher levels of both tissue inhibitor of metalloproteinases 1 (TIMP‐1; 364 ng/mL vs. former: 324 ng/mL vs. never: 278 ng/mL; *p* < 0.001) and procollagen type III N‐terminal propeptide (P3NP; 20.9 μg/L vs. former: 15.6 μg/L vs. never: 17.2 μg/L; *p* = 0.008) (Table 1, Figure S2). After adjusting for MELD and HVPG, active smokers showed significantly higher ELF (*p* = 0.027) and TIMP‐1 (*p* < 0.001) levels compared to never‐smokers, while no significant differences were observed between former and never‐smokers. P3NP levels did not significantly differ by smoking status. ELF components did not differ significantly in active or former smokers with < 30 versus ≥ 30 PY (Table S1).

Within the analysis of parameters reflecting pathological angiogenesis, active smokers showed significantly higher levels of placental growth factor (PLGF; 21.7 pg/mL) than former (18.2 pg/mL) or never‐smokers (19.0 pg/mL; *p* = 0.004). After adjusting for MELD and HVPG, active smokers showed significantly higher PLGF levels (*p* < 0.001) compared to never‐smokers, while no significant differences were observed between former and never smokers. For soluble vascular endothelial growth factor‐receptor 1 (sVEGFR1), a scavenger of PLGF, no significant differences were observed (active: 107 pg/mL vs. former: 103 pg/mL vs. never 106 pg/mL; *p* = 0.847) (Table 1, Figure S2). However, after stratifying the cohort according to PY, patients with ≥ 30 PY not only showed significantly higher levels of PLGF (21.7 pg/mL vs. < 30 PY: 18.7 pg/mL vs. never‐smokers: 19.0 pg/mL; *p* = 0.010) but also of sVEGFR1 (115 pg/mL vs. < 30 PY: 95 pg/mL vs. never‐smokers 106 pg/mL; *p* = 0.046) (Table S1).

### 3.3 Hepatocellular Carcinoma and Extrahepatic Malignancies

**FIGURE 1:**
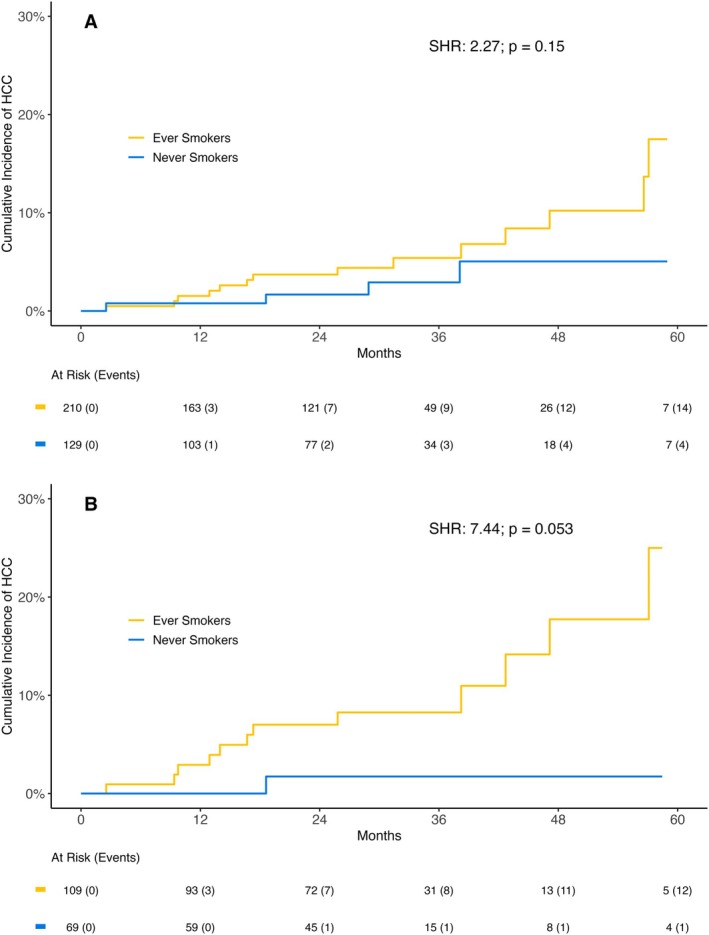
Cumulative incidence of hepatocellular carcinoma in never smokers compared to ever smokers in (A) the complete study cohort and (B) Child‐Pugh A patients. Abbreviations: HCC, hepatocellular carcinoma; SHR, subdistribution hazard ratio.

Over a median follow‐up of 30.4 (21.2–44.6) months, 18 patients developed de novo HCC—4 occurred in never‐smokers and 14 in ever‐smokers, with 6 cases in former smokers and 8 in active smokers. Accordingly, in the univariable competing risk regression model (Table S2, Figure 1A), ever‐smokers showed a nonsignificant trend toward a higher risk of de novo HCC when compared to never‐smokers (subdistribution hazard ratio [SHR]: 2.27; 95% confidence interval [CI]: 0.75–6.89; *p* = 0.150). In a subgroup analysis of Child‐Pugh A patients (*n* = 178), there was a trend toward higher risk in ever‐smokers (1/69 never‐smokers [1.4%] vs. 12/109 ever‐smokers [11.0%]; SHR 7.44 (95% CI: 0.97–56.90; *p* = 0.053) (Figure 1B)).

In the multivariable competing risk regression models (Table S2) adjusted for viral aetiology, smoking remained numerically associated with a higher incidence for HCC with an aSHR 2.15 (*p* = 0.180).

There was neither an association of HCC occurrence with the number of PY in ever‐smokers (SHR per PY: 0.99; 95% CI: 0.97–1.01; *p* = 0.570; Table S3) or active smokers (SHR per PY: 1.00; 95% CI: 0.96–1.04; *p* = 0.880).

Extrahepatic malignancies occurred in 18 patients during follow‐up, with one case in a never‐smoker, nine in former smokers and eight in active smokers. Among the 26 patients who underwent liver transplantation within our cohort, no de novo extrahepatic malignancy was observed during their follow‐up period. The only extrahepatic malignancy in never‐smokers was a cholangiocarcinoma, which developed in a patient with primary sclerosing cholangitis. In contrast, the majority of the 17 de novo extrahepatic malignancies that occurred in ever‐smokers were found in the respiratory tract (*n* = 3 lung cancer, *n* = 1 cancer of the vocal folds), the oropharynx and tongue (*n* = 3), the bladder (*n* = 2) and the rectum (*n* = 2).

**FIGURE 2:**
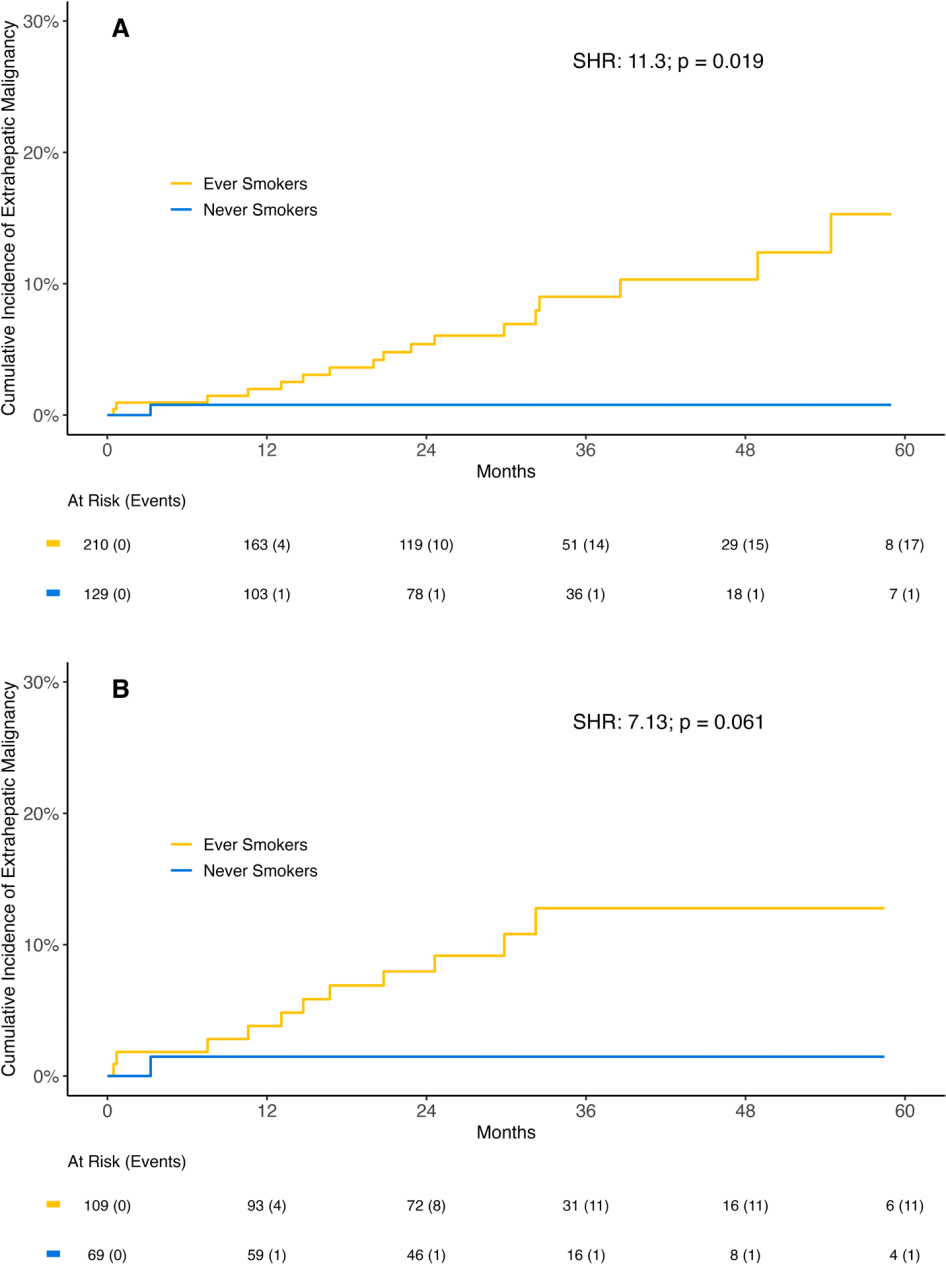
Cumulative incidence of extrahepatic malignancy in never smokers compared to ever smokers in (A) the complete study cohort and (B) Child‐Pugh A patients. Abbreviations: SHR, subdistribution hazard ratio.

In the univariable competing risk regression model, ever‐smokers had a significantly higher risk of de novo extrahepatic malignancies compared to never‐smokers, both in the overall cohort (SHR: 11.30; 95% CI: 1.49–85.90; *p* = 0.019) (Table 2, Figure 2A) and in the subgroup of Child‐Pugh A patients (SHR: 7.13; 95% CI: 0.92–55.40; *p* = 0.061) (Figure 2B). In the multivariable models (Table 2) adjusted for age and MELD, smoking remained significantly associated with a higher risk for extrahepatic malignancies (aSHR: 11.03; *p* = 0.021).

**TABLE 2:** Smoking and risk of extrahepatic malignancy using competing risk regression analysis.

	Univariable analysis			Multivariable model 1 adjusted for MELD			Multivariable model 2 adjusted for diabetes			Multivariable model 3 adjusted for ALD		
	SHR	95% CI	*p*	aSHR	95% CI	*p*	aSHR	95% CI	*p*	aSHR	95% CI	*p*
Ever smoking, yes	11.30	1.49–85.90	0.019	11.03	1.43–84.31	0.021	10.82	1.43–81.99	0.021	10.72	1.42–81.23	0.022
Age, per year	1.04	1.00–1.09	0.052	1.05	1.00–1.10	0.076	1.04	0.99–1.10	0.120	1.04	0.99–1.10	0.094
MELD, per point	0.97	0.87–0.1.08	0.520	0.97	0.86–1.06	0.410						
Diabetes, present	1.50	0.57–3.98	0.410				1.13	0.39–3.26	0.830			
ALD, vs. other aetiologies	1.48	0.59–3.74	0.410							1.22	0.49–3.05	0.670

*Note:* Uni‐ and multivariable Fine–Gray competing risk regression models assessing predictors of de novo extrahepatic malignancy with death and liver transplantation as competing events. Follow‐up was censored at the earliest occurrence of liver transplantation or death.

Abbreviations: ALD, alcohol‐related liver disease; aSHR, adjusted subdistribution hazard ratio; MELD, model for end‐stage liver disease; SHR, subdistribution hazard ratio.

With regard to the impact of PY, there was no association with extrahepatic malignancies in ever‐smokers (SHR per PY: 1.00; 95% CI: 0.99–1.02; *p* = 0.770; Table S4) or active smokers (aSHR per PY: 1.00; 95% CI: 0.96–1.03; *p* = 0.890) in a multivariable model including age. Furthermore, in former smokers, the risk did not differ significantly when compared to active smokers (SHR former vs. active: 1.67; 95% CI: 0.67–4.24; *p* = 0.280) and the duration of being smoke‐free was not associated with a decreased risk (SHR per year: 1.01; 95% CI: 0.99–1.03; *p* = 0.430).

### 3.4 Major Adverse Cardiovascular Events and Mortality

At baseline, the proportion of patients with preexisting coronary artery disease and prior MACE was significantly higher in ever‐smokers when compared to never‐smokers with 16.2% versus 2.3% for coronary artery disease (*p* < 0.001) and 7.1% versus 2.3% for prior MACE (*p* = 0.055). During follow‐up, nine patients experienced MACE including *n* = 3 myocardial infarctions, *n* = 2 strokes and *n* = 4 cardiovascular deaths, with two and seven MACE occurring in never‐smokers and in ever‐smokers (*n* = 4 in former smokers; *n* = 3 in active smokers) respectively. Ever‐smokers had a numerically higher risk of MACE when compared to never‐smokers (SHR: 2.18; 95% CI: 0.46–10.40; *p* = 0.330), which remained true after including preexisting coronary artery disease in a multivariable model (SHR: 1.93; 95% CI: 0.40–9.38; *p* = 0.420). Furthermore, the number of PY in ever‐smokers was linked to a numerically higher risk of MACE (SHR per PY: 1.01; 95% CI: 0.99–1.03; *p* = 0.470).

During the study period, 80 patients died, with 55 of these deaths being liver‐related. In never and ever‐smokers, 28 (21.7%; *n* = 21 liver‐related) and 52 (24.8%; *n* = 34 liver related) died respectively. In the univariable competing risk regression model, ever‐smokers did not show a significantly higher risk of all‐cause mortality when compared to never‐smokers, both in the overall cohort (SHR: 1.18; 95% CI: 0.75–1.86; *p* = 0.490) and in the subgroup of Child‐Pugh A patients (SHR: 1.36; 95% CI: 0.64–2.87; *p* = 0.430). Both in ever‐smokers (SHR per PY: 1.00; 95% CI: 0.99–1.01; *p* = 0.790) and active smokers (SHR per PY: 0.99; 95% CI: 0.97–1.01; *p* = 0.300), the number of PY was not significantly linked to an altered risk of all‐cause mortality and the duration of being smoke‐free was not associated with a decreased risk (SHR per year: 0.99; 95% CI: 0.97–1.01; *p* = 0.420). Similarly, ever‐smokers did not have a higher risk of liver‐related mortality when compared to never‐smokers (SHR: 1.00; 95% CI: 0.58–1.72; *p* = 1.000) and the number of PY in ever‐smokers was not linked to a higher risk (SHR per PY: 1.00; 95% CI: 0.99–1.01; *p* = 0.920).

## 4 Discussion

Despite the widely known risks associated with tobacco smoking, its role in ACLD remains understudied. Smoking has negative effects on innate and adaptive immunity [30], both of which are already impaired in ACLD due to cirrhosis‐associated immune dysfunction, as characterised by systemic inflammation and compromised immune function [31]. In our study, active smokers exhibited significantly higher WBC and CRP levels compared to former and never‐smokers. Furthermore, the presence of elevated LBP levels in smokers indicates increased bacterial translocation, which has been previously linked to hepatic fibrogenesis [32] and liver disease progression in ACLD [33, 34]. Thus, smoking may further increase the already elevated risk of bacterial infections in ACLD patients, which can significantly worsen the prognosis [34, 35].

The role of smoking in promoting fibrogenesis is well supported by experimental evidence [10, 11], yet clinical data obtained from ACLD patients are scarce. In our study, active smokers exhibited significantly higher levels of TIMP‐1 and P3NP compared to former and never‐smokers, indicating a link between active smoking and increased fibrogenesis. Additionally, ELF test levels progressively increased from never‐smokers to former and active smokers. Potential underlying mechanisms for smoking‐induced fibrogenesis include transforming growth factor‐beta‐1 release [35], an increase in proinflammatory cytokines [36] and reactive oxygen species production [26], all of which promote fibroblast activation and an increased production of extracellular matrix proteins [10, 11]. Interestingly, despite the evidence of enhanced fibrogenic activity in active smokers through elevated serum fibrogenesis markers, HVPG and liver stiffness were similar across smoking groups. The lack of correlation in surrogates of (static) fibrosis severity on cross‐sectional assessment does, however, not preclude that smoking triggers (active) fibrogenic processes and thereby impacts on long‐term liver disease progression.

In addition, smoking was associated with increased levels of PLGF in our cohort, indicating enhanced angiogenic activity. PLGF, a member of the vascular endothelial growth factor family, is involved in pathological angiogenesis by enhancing endothelial cell proliferation and the growth and maturation of blood vessels [37]. Angiogenesis plays a critical role in liver disease progression [38] and is essential in the development and progression of HCC, a prototypical hypervascular tumour [39].

Regarding malignancies, our study found a significant association between smoking and extrahepatic malignancies in ACLD patients. While we observed a numerical increase in HCC incidence among ever‐smokers compared to never‐smokers, this association did not reach statistical significance in our cohort. This trend aligns with previous meta‐analyses and cohort studies that have identified smoking as a risk factor for HCC (7–9), though our study may have been underpowered to detect a significant difference. The strongest evidence from our study relates to the effects of smoking on inflammatory, fibrogenic and angiogenic pathways, which may contribute to cancer risk in the long term.

Furthermore, our study observed a significant association between viral hepatitis and HCC development. This association persists even though a substantial proportion of our viral hepatitis patients (*n* = 39, 69.6%) had achieved SVR prior to enrolment. This highlights that SVR does not eliminate the risk of HCC, particularly in patients who have already progressed to ACLD [40]. The persistent HCC risk after viral eradication, thus, continues to contribute to the observed association between viral aetiology and HCC in our ACLD cohort.

Additionally, smoking was associated with a higher incidence of extrahepatic malignancies, including cancers of the respiratory and urinary tract. These results highlight the broad carcinogenic potential of smoking, impacting both hepatic and extrahepatic sites. It is also important to consider the concept of latency, particularly in former smokers. While smoking cessation is undoubtedly beneficial, the precise kinetics of the regression of smoking‐induced detrimental effects in ACLD remain complex. Our data show no significant correlation between the duration of smoking cessation and improvements in markers of inflammation, fibrogenesis or angiogenesis among former smokers. This observation can be interpreted in two ways: Either the pro‐inflammatory, pro‐fibrogenic and pro‐angiogenic effects persist for a considerable time, implying a delayed reversibility, or conversely, the most substantial benefits occur relatively quickly after cessation, making further incremental improvements with prolonged abstinence less discernible in this advanced disease setting. Indeed, the broader carcinogenic potential of toxicants, including those from cigarette smoke, is further highlighted by a prospective study [41] showing long‐term occupational toxicant exposure linked to increased ACLD/HCC risk. Both interpretations underscore the critical importance of early and sustained smoking cessation in ACLD patients, warranting future studies with longer follow‐up.

Interestingly, in our cohort, ever‐smokers did not show a significantly increased risk of all‐cause or liver‐related mortality. This may reflect that ACLD itself, as a severe and progressive disease, often represents the main determinant of clinical outcomes, thereby narrowing the ‘detrimental impact window’ for smoking's additional impact. Further explanations include our cohort's advanced disease state, a potentially insufficient median 30.4‐month follow‐up and the high comorbidity prevalence that might dilute smoking's relative mortality contribution.

While our findings are based on a thoroughly characterised cohort of ACLD patients, some limitations need to be acknowledged: First, the reliance on self‐reported smoking data without biochemical verification (e.g., cotinine levels) may introduce bias. Nevertheless, all patients without sufficient information on smoking status and PY were excluded, thus limiting the impact of this limitation. Second, other lifestyle factors such as physical activity and diet were not documented, which could confound the results. Third, the absence of observed dynamic changes in smoking status during the follow‐up period is a limitation. Interestingly, our analysis did not identify male sex as a significant risk factor for HCC development despite its well‐established role in hepatocarcinogenesis [42]. This discrepancy likely stems from our limited sample size, the pre‐selection of an ACLD cohort or the predominance of alcohol‐related aetiology. Lastly, our cohort only included a comparably low number of patients with low PY exposure, thus limiting any potential analyses regarding the impact of low‐level smoking.

Given the detrimental effects of smoking on inflammation, fibrogenesis, angiogenesis and cancer risk, smoking cessation should be a priority in the management of ACLD patients. Integrating smoking cessation programmes into routine patient care could not only significantly reduce disease progression and cancer rates but also improve clinical outcomes and survival. Moreover, addressing smoking‐related risks in ACLD patients through targeted therapies and lifestyle interventions may further enhance their overall health and quality of life.

In conclusion, smoking significantly aggravates inflammation, fibrogenesis and angiogenesis in patients with ACLD, and is associated with an increased risk of extrahepatic malignancies. These biological mechanisms are linked to hepatocarcinogenesis and thus likely contribute to hepatic cancer risk, though this association did not reach statistical significance in our cohort. Our findings emphasise the need for robust smoking cessation efforts and comprehensive lifestyle management in the care of liver disease patients.

## Author Contributions

All authors contributed either to the study concept and design (N.D., T.R. and B.S.H.) and/or data acquisition (all authors), analysis (N.D., T.R. and B.S.H.) or interpretation (all authors). N.D., T.R. and B.S.H. drafted the manuscript, which was critically revised by all other authors. All authors read and approved the final manuscript.

## Disclosure

**N.D**. received travel support from Gilead. **B.Si**. received travel support from AbbVie, Gilead and Falk. **B.Sc**. received travel support from AbbVie, Ipsen and Gilead. **M.S**. received travel support from MSD, Sandoz, BMS, AbbVie and Gilead; and speaking honoraria from BMS and Gilead. **L.H**. has nothing to disclose. **M.J**. served as speaker and/or consultant for Gilead. **L.B**. received speaking fees from Chiesi, and Gilead. **G.S**. received travel support from Amgen. **G.K**. has nothing to disclose. **C.S**. has nothing to disclose. **M.T**. received grant support from Albireo, Alnylam, Cymabay, Falk, Genentech, Gilead, Intercept, MSD, Takeda and UltraGenyx, honoraria for consulting from AbbVie, Albireo, Agomab, Boehringer Ingelheim, BiomX, Chemomab, Dexoligo Therapeutics, Falk, Genfit, Gilead, GSK, Hightide, Intercept, Ipsen, Jannsen, MSD, Novartis, Phenex, Pliant, Regulus, Siemens and Shire, speaker fees from Albireo, Boehringer Ingelheim, Bristol‐Myers Squibb, Falk, Gilead, Ipsen, Intercept, MSD and Madrigal, as well as travel support from AbbVie, Falk, Gilead, Jannsen and Intercept. He is also co‐inventor of patents on the medical use of 24‐norursodeoxycholic acid filed by the Medical University of Graz. **M.P**. received speaker honoraria from Bayer, BMS, Eisai, Ipsen, Lilly, MSD and Roche; he is a consultant/advisory board member for AstraZeneca, Bayer, BMS, Eisai, Ipsen, Lilly, MSD and Roche; he received grants from AstraZeneca, Bayer, BMS, Eisai and Roche; he received travel support from Bayer, BMS, Ipsen and Roche. **M.M**. served as a speaker and/or consultant and/or advisory board member for AbbVie, Collective Acumen, Gilead, Takeda and W. L. Gore & Associates and received travel support from AbbVie and Gilead. **T.R**. received grant support from AbbVie, Boehringer‐Ingelheim, Gilead, Intercept, MSD, Myr Pharmaceuticals, Philips Healthcare, Pliant, Siemens and W. L. Gore & Associates; speaking honoraria from AbbVie, Gilead, Gore, Intercept, Roche and MSD; consulting/advisory board fees from AbbVie, Bayer, Boehringer‐Ingelheim, Gilead, Intercept, MSD and Siemens; and travel support from AbbVie, Boehringer‐Ingelheim, Gilead and Roche. **B.S.H**. received travel support from Ipsen and Falk.

## Supporting information


**Figure S1:** liv70314‐sup‐0001‐FigureS1.pdf.

**Figure S2:** liv70314‐sup‐0002‐FigureS2.pdf.

**Data S1:** liv70314‐sup‐0003‐DataS1.docx.

## Data Availability

The data that support the findings of this study are available from the corresponding author upon reasonable request.
