# Supplementary material for: Smoking Aggravates Inflammation, Fibrogenesis, Angiogenesis and Cancer Risk in Patients With Cirrhosis
Source: Liver Int. 2025 Sep 3;45(10):e70314. doi: 10.1111/liv.70314 (PMC12406090; doi:10.1111/liv.70314)
Supplement: Supplementary file 1 — Figure S1: liv70314‐sup‐0001‐FigureS1.pdf. [file LIV-45-0-s002.pdf]

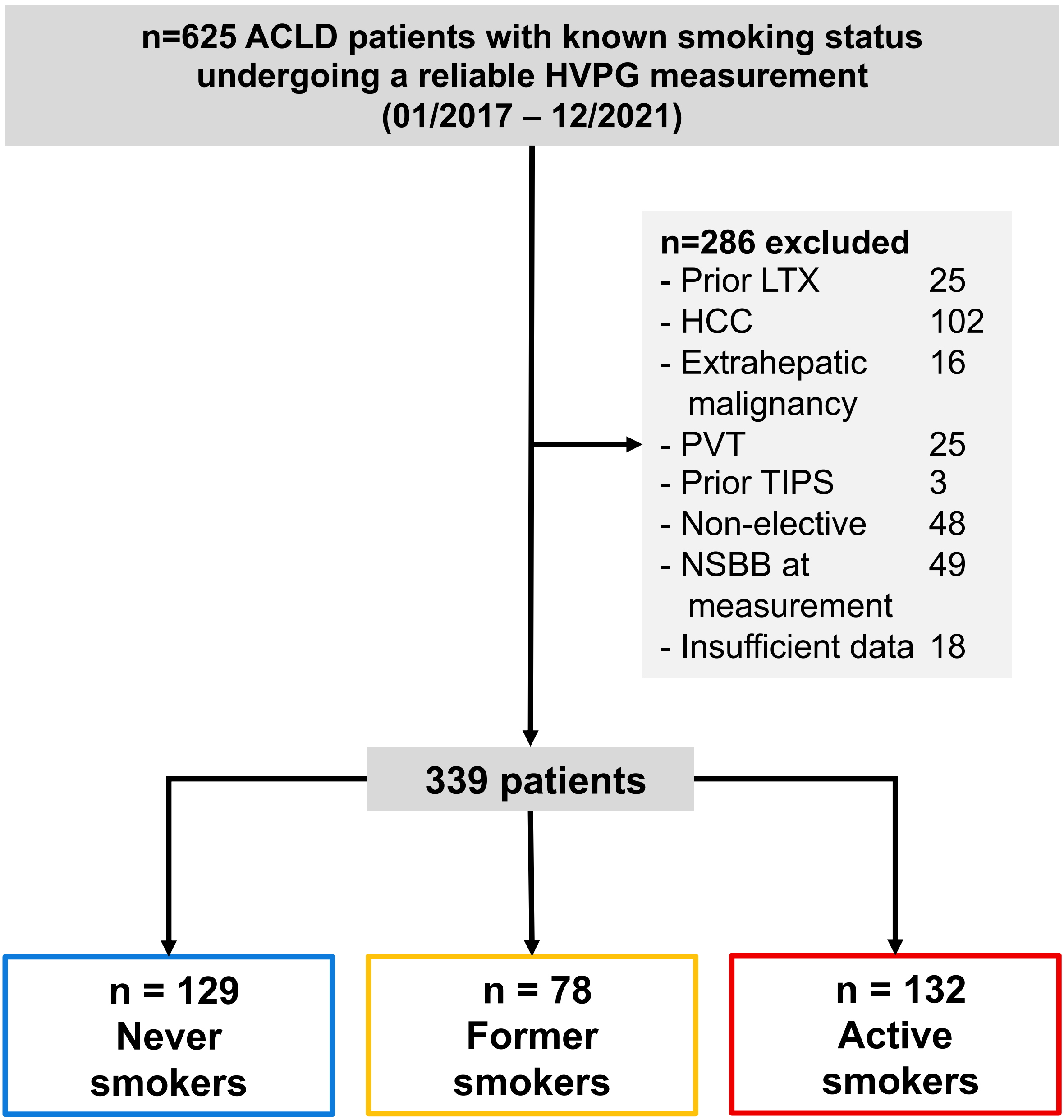
n=625 ACLD patients with known smoking status undergoing a reliable HVPG measurement (01/2017 – 12/2021)
n=286 excluded
- Prior LTX 25
- HCC 102
- Extrahepatic malignancy 16
- PVT 25
- Prior TIPS 3
- Non-elective 48
- NSBB at measurement 49
- Insufficient data 18
339 patients
n = 129 Never smokers
n = 78 Former smokers
n = 132 Active smokers
