# Supplementary figures and images for: Smoking Aggravates Inflammation, Fibrogenesis, Angiogenesis and Cancer Risk in Patients With Cirrhosis
Source: Liver Int. 2025 Sep 3;45(10):e70314. doi: 10.1111/liv.70314 (PMC12406090; doi:10.1111/liv.70314)

**Inflammation and bacterial translocation**

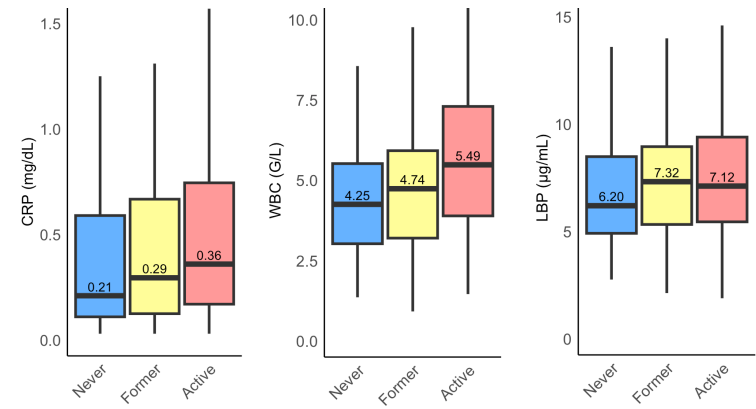

**Fibrogenesis**

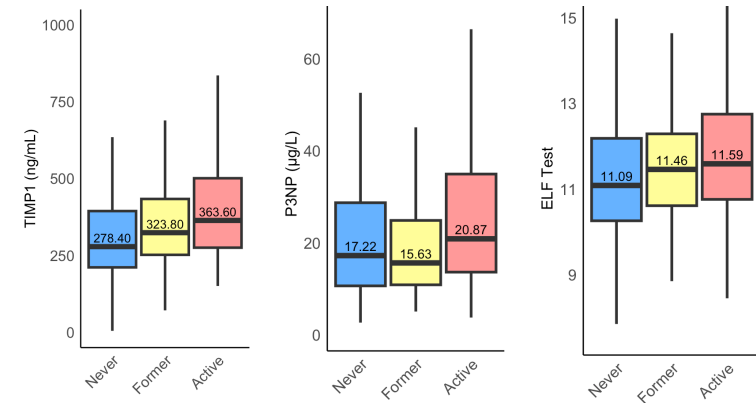

**Angiogenesis**

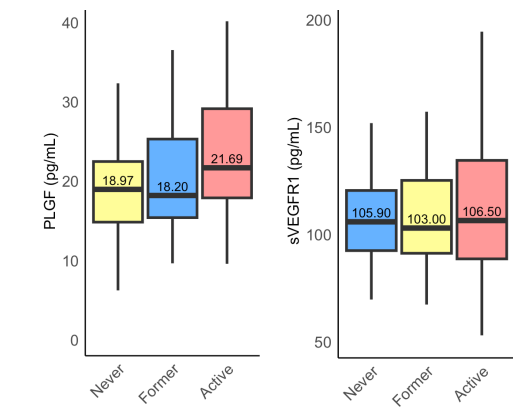

Supplement: Supplementary file 2 — Figure S2: liv70314‐sup‐0002‐FigureS2.pdf. [file LIV-45-0-s003.pdf]
