# Supplementary material for: Smoking Aggravates Inflammation, Fibrogenesis, Angiogenesis and Cancer Risk in Patients With Cirrhosis
Source: Liver Int. 2025 Sep 3;45(10):e70314. doi: 10.1111/liv.70314 (PMC12406090; doi:10.1111/liv.70314)
Supplement: Supplementary file 3 — Data S1: liv70314‐sup‐0003‐DataS1.docx. [file LIV-45-0-s001.docx]

SUPPLEMENTARY MATERIAL

***Impact of smoking on biomarkers stratified by ALD and adjusted for alcohol consumption***

To address the potential confounding effect of ALD and ongoing alcohol consumption during follow up, we performed stratified analyses. When restricting the analysis to non-ALD patients (n=181), active smokers still showed significantly higher levels of WBC (5.46 vs. former: 4.83 vs. never: 3.71 G/L; p=0.007), CRP (0.25 vs. former: 0.27 vs. never: 0.15 mg/dL; p=0.021), LBP (7.12 vs. former: 7.44 vs. never: 5.92; p=0.015), and TIMP-1 (322 vs. former: 281 vs. never: 247 ng/mL; p=0.003). PLGF, ELF, P3NP and sVEGFR1 were not significantly different between groups in this stratified analysis. In multivariable models adjusting for MELD, HVPG, ALD, and ongoing alcohol consumption during follow-up, active smokers consistently showed significantly higher levels of WBC (adjusted p=0.001), CRP (adjusted p=0.004), TIMP-1 (adjusted p<0.001), and PLGF (adjusted p<0.003) compared to never-smokers. LBP levels, ELF, and P3NP levels did not show significant differences in these fully adjusted models.

***Correlation between Duration of Smoking Cessation and Biomarkers in Former Smokers***

In former smokers, there was no significant correlation between the duration free of smoking and WBC (Spearman’s rho: -0.01; p=0.931), CRP (r:0.04; p=0.737) or LBP (r:0.09; p=0.422). There was no significant correlation of ELF (r:-0.14; p=0.235), TIMP-1 (r:-0.04; p=0.709), P3NP (r:-0.07; p=0.536) or hyaluronic acid (HA; r:-0.14; p=0.235) and the duration free of smoking in former smokers and the time free of smoking did not correlate with PLGF (r:0.08; p=0.639) or sVEGFR1 (r:0.00; p=0.990) levels.

Supplementary Table S1. Patient characteristics stratified by packyears.

|  | **Never**  **smokers**  **n=129** | **<30**  **packyears**  **n=97** | **≥30**  **packyears**  **n=113** | **p-value** |
| --- | --- | --- | --- | --- |
| **Age, years** | 54.0 (46.5-65.2)^§^ | 55.4 (43.3-61.3)^§^ | 59.7 (52.9-67.1)*^†^ | **0.002** |
| **Sex, male** | 70 (54.3%) | 64 (66.0%) | 90 (79.6%) | **<0.001** |
| **Aetiology** |  |  |  | **<0.001** |
| **ALD** | 50 (38.8%) | 45 (46.4%) | 63 (55.8%) |  |
| **Viral** | 23 (17.8%) | 16 (16.5%) | 17 (15.0%) |  |
| **ALD/Viral** | 0 (0%) | 8 (8.2%) | 13 (11.5%) |  |
| **MASLD** | 14 (10.9%) | 7 (7.2%) | 9 (8.0%) |  |
| **Cholestatic** | 13 (10.1%) | 7 (7.2%) | 0 (0%) |  |
| **Other** | 29 (22.5%) | 14 (14.4%) | 11 (9.7%) |  |
| **HVPG, mmHg** | 16 (11-20) | 18 (11-22) | 17 (13-20) | 0.561 |
| **Compensated** | 61 (47.3%) | 32 (33.0%) | 39 (34.5%) | **0.046** |
| **MELD** | 11 (8-15) | 11 (9-16) | 10 (8-15) | 0.572 |
| **Child-Pugh stage** |  |  |  | 0.886 |
| **A** | 69 (53.5%) | 49 (50.5%) | 60 (53.1%) |  |
| **B** | 48 (37.2%) | 35 (36.1%) | 39 (34.5%) |  |
| **C** | 12 (9.3%) | 13 (13.4%) | 14 (12.4%) |  |
| **Diabetes** | 35 (27.1%) | 24 (24.7%) | 28 (24.8%) | 0.889 |
| **Art. hypertension** | 45 (34.9%) | 25 (25.8%) | 39 (34.5%) | 0.302 |
| **Albumin, g/L** | 36.8 (32.8-40.2) | 37.0 (32.9-40.2) | 36.3 (32.4-40.2) | 0.748 |
| **Bilirubin, mg/dL** | 1.12 (0.75-1.95) | 1.10 (0.78-2.37)^§^ | 0.92 (0.65-1.74)^†^ | **0.018** |
| **INR** | 1.3 (1.2-1.6) | 1.4 (1.2-1.5) | 1.3 (1.2-1.5) | 0.630 |
| **Creatinine, mg/dL** | 0.73 (0.59-0.90) | 0.72 (0.58-0.86)^§^ | 0.81 (0.63-1.02)^†^ | **0.023** |
| **Sodium, mmol/L** | 139 (137-141) | 138 (136-140) | 138 (136-141) | 0.139 |
| **Platelets, G/L** | 100 (76-132) | 103 (71-141) | 106 (76-160) | 0.500 |
| **WBC, G/L** | 4.26 (3.03-5.52)^†§^ | 5.27 (3.37-6.81)* | 4.99 (3.48-6.61)* | **0.011** |
| **CRP, mg/dL** | 0.21 (0.11-0.59) | 0.33 (0.12-0.76) | 0.36 (0.17-0.68) | **0.044** |
| **IL-6, mg/dL** | 9.61 (4.73-17.20) | 8.32 (5.19-15.00) | 10.30 (5.85-21.60) | 0.153 |
| **LBP, µg/mL** | 6.20 (4.92-8.49) | 7.10 (5.43-9.05) | 7.30 (5.32-9.44) | 0.063 |
| **vWF Antigen, %** | 264 (205-333) | 270 (207-352) | 300 (215-372) | 0.424 |
| **AST, U/L** | 43 (32-59) | 47 (30-59) | 40 (28-54) | 0.228 |
| **ALT, U/L** | 32 (23-46) | 31 (21-43) | 27 (19-41) | 0.082 |
| **ELF** | 11.1 (10.3-12.2) | 11.7 (10.7-12.4) | 11.5 (10.8-12.7) | 0.140 |
| **TIMP-1, ng/mL** | 278 (211-394)^§^ | 325 (252-474) | 369 (276-493)* | **0.001** |
| **P3NP, μg/L** | 17.2 (10.6-28.7) | 21.2 (12.9-31.4) | 17.8 (11.6-30.2) | 0.162 |
| **HA, ng/ml** | 179 (96-365) | 243 (106-463) | 234 (125-546) | 0.279 |
| **sVEGFR1, pg/mL** | 106 (93-121) | 95 (87-118) | 115 (95-134) | **0.046** |
| **PLGF, pg/mL** | 19.0 (14.8-22.5)^§^ | 18.7 (16.5-25.0) | 21.7 (18.2-30.3)* | **0.010** |

Data presented as number n (%) or median (IQR). Data on TIMP-1, P3NP and HA as components of the ELF test are missing in n=20 patients. Data on SFLT1 (sVEGFR1) and PLGF are missing in n=153 patients. Abbreviations: ALD, alcohol-related liver disease; MASLD, metabolic-dysfunction associated steatotic liver disease; HVPG, hepatic venous pressure gradient; MELD, model for end-stage liver disease; INR, international normalised ration; WBC, white blood cell count; CRP, C-reactive protein; IL-6, interleukin 6; LBP, lipopolysaccharide binding protein; vWF, von Willebrand factor; AST, aspartate aminotransferase; ALT, alanine aminotransferase; ELF enhanced liver fibrosis test; TIMP-1, tissue inhibitor of metalloproteinases 1; P3NP, procollagen type III N-terminal propeptide; HA, hyaluronic acid; sVEGFR1, soluble vascular endothelial growth factor receptor 1; PLGF, placental growth factor. P-values in bold indicate statistical significance. *p<0.05 when compared to never smokers, † vs. <30 packyears, § vs. ≥30 packyears.

Supplementary Table S2. Smoking and risk of hepatocellular carcinoma using competing risk regression analysis

|  | **Univariable analysis** | | | **Multivariable model 1**  Including MELD and HVPG | | | **Multivariable model 2**  Including Age and Sex | | | **Multivariable model 3**  Including Diabetes and Viral aetiology | | |
| --- | --- | --- | --- | --- | --- | --- | --- | --- | --- | --- | --- | --- |
|  | **SHR** | **95% CI** | **p-value** | **aSHR** | **95% CI** | **p-value** | **aSHR** | **95% CI** | **p-value** | **aSHR** | **95% CI** | **p-value** |
| **Ever smoking, yes** | 2.27 | 0.75-6.89 | 0.150 | 2.30 | 0.75-7.07 | 0.150 | 2.28 | 0.81-6.44 | 0.120 | 2.15 | 0.71-6.54 | 0.180 |
| **MELD, per point** | 0.97 | 0.90-1.05 | 0.460 | 0.97 | 0.79-1.07 | 0.560 |  |  |  |  |  |  |
| **HVPG, per mmHg** | 0.99 | 0.91-1.07 | 0.740 | 0.99 | 0.90-1.09 | 0.880 |  |  |  |  |  |  |
| **Age, per year** | 1.01 | 0.97-1.04 | 0.790 |  |  |  | 1.00 | 0.96-1.04 | 0.890 |  |  |  |
| **Sex, male** | 1.06 | 0.40-2.86 | 0.910 |  |  |  | 0.95 | 0.36-2.48 | 0.910 |  |  |  |
| **Diabetes, present** | 0.82 | 0.27-2.46 | 0.720 |  |  |  |  |  |  | 0.67 | 0.21-2.13 | 0.500 |
| **Viral, vs. other aetiologies** | 4.31 | 1.72-10.80 | 0.002 |  |  |  |  |  |  | 4.37 | 1.64-11.65 | 0.003 |

Uni- and multivariable Fine-Gray competing risk regression models assessing predictors of de novo HCC with death and liver transplantation as competing events. Follow-up was censored at the earliest occurrence of liver transplantation, or death. Abbreviations: MELD, model for end-stage liver disease; HVPG, hepatic venous pressure gradient; ALD, alcohol-related liver disease.

Supplementary Table S3. Pack years and risk of hepatocellular carcinoma using competing risk regression analysis

|  | **Univariable analysis** | | | **Multivariable model 1**  adjusted for MELD and HVPG | | | **Multivariable model 2**  adjusted for Age and Sex | | | **Multivariable model 3**  adjusted for Diabetes and Viral aetiology | | |
| --- | --- | --- | --- | --- | --- | --- | --- | --- | --- | --- | --- | --- |
|  | **SHR** | **95% CI** | **p-value** | **aSHR** | **95% CI** | **p-value** | **aSHR** | **95% CI** | **p-value** | **aSHR** | **95% CI** | **p-value** |
| **Pack years** | 0.99 | 0.97-1.01 | 0.570 | 1.00 | 0.97-1.02 | 0.600 | 0.99 | 0.97-1.01 | 0.190 | 1.00 | 0.98-1.02 | 0.870 |
| **MELD, per point** | 0.97 | 0.90-1.05 | 0.460 | 0.94 | 0.84-1.06 | 0.300 |  |  |  |  |  |  |
| **HVPG, per mmHg** | 0.99 | 0.91-1.07 | 0.740 | 0.98 | 0.89-1.07 | 0.610 |  |  |  |  |  |  |
| **Age, per year** | 1.01 | 0.97-1.04 | 0.790 |  |  |  | 1.04 | 0.99-1.09 | 0.160 |  |  |  |
| **Sex, male** | 1.06 | 0.40-2.86 | 0.910 |  |  |  | 1.58 | 0.44-5.69 | 0.490 |  |  |  |
| **Diabetes, present** | 0.82 | 0.27-2.46 | 0.720 |  |  |  |  |  |  | 0.93 | 0.26-3.32 | 0.920 |
| **Viral, vs. other aetiologies** | 4.31 | 1.72-10.80 | 0.002 |  |  |  |  |  |  | 3.76 | 1.23-11.51 | 0.020 |

Uni- and multivariable Fine-Gray competing risk regression models assessing predictors of de novo HCC with death and liver transplantation as competing events. Follow-up was censored at the earliest occurrence of liver transplantation, or death. Abbreviations: MELD, model for end-stage liver disease; HVPG, hepatic venous pressure gradient; ALD, alcohol-related liver disease.

Supplementary Table S4. Pack years and risk of extrahepatic malignancy using competing risk regression analysis

|  | **Univariable analysis** | | | **Multivariable model 1**  adjusted for MELD | | | **Multivariable model 2**  adjusted for Diabetes | | | **Multivariable model 3**  adjusted for ALD | | |
| --- | --- | --- | --- | --- | --- | --- | --- | --- | --- | --- | --- | --- |
|  | **SHR** | **95% CI** | **p-value** | **aSHR** | **95% CI** | **p-value** | **aSHR** | **95% CI** | **p-value** | **aSHR** | **95% CI** | **p-value** |
| **Pack years** | 1.00 | 0.99-1.02 | 0.770 | 0.99 | 0.97-1.01 | 0.310 | 0.99 | 0.97-1.01 | 0.310 | 0.99 | 0.97-1.01 | 0.290 |
| **Age, per year** | 1.04 | 1.00-1.09 | 0.052 | 1.07 | 1.02-1.12 | 0.003 | 1.07 | 1.02-1.13 | 0.010 | 1.07 | 1.02-1.12 | 0.005 |
| **MELD, per point** | 0.97 | 0.87-.1.08 | 0.520 | 0.96 | 0.89-1.07 | 0.500 |  |  |  |  |  |  |
| **Diabetes, present** | 1.50 | 0.57-3.98 | 0.410 |  |  |  | 1.12 | 0.37-3.44 | 0.840 |  |  |  |
| **ALD, vs. other aetiologies** | 1.48 | 0.59-3.74 | 0.410 |  |  |  |  |  |  | 1.41 | 0.53-3.75 | 0.490 |

Uni- and multivariable Fine-Gray competing risk regression models assessing predictors of de novo extrahepatic malignancy with death and liver transplantation as competing events. Follow-up was censored at the earliest occurrence of liver transplantation, or death. Abbreviations: SHR, subdistribution hazard ratio; aSHR, adjusted subdistribution hazard ratio; MELD, model for end-stage liver disease; ALD, alcohol-related liver disease**.**

Supplementary Fig. S1. Study patient selection process.

Abbreviations: ACLD, advanced chronic liver disease; HVPG, hepatic venous pressure gradient; LTX, liver transplantation; HCC, hepatocellular carcinoma; PVT, portal vein thrombosis; TIPS, transjugular intrahepatic portosystemic shunt; NSBB, non-selective betablocker.

Supplementary Fig. S2. Boxplots of biomarkers associated with systemic inflammation and bacterial translocation, fibrogenesis and angiogenesis stratified by smoking status (Never, Former, Active).

Abbreviations: WBC, white blood cell count; CRP, C-reactive protein; LBP, lipopolysaccharide binding protein; ELF enhanced liver fibrosis test; TIMP-1, tissue inhibitor of metalloproteinases 1; P3NP, procollagen type III N-terminal propeptide;; sVEGFR1, soluble vascular endothelial growth factor receptor 1; PLGF, placental growth factor.
